# Engineering Specificity and Function of Therapeutic Regulatory T Cells

**DOI:** 10.3389/fimmu.2017.01517

**Published:** 2017-11-10

**Authors:** Jenny L. McGovern, Graham P. Wright, Hans J. Stauss

**Affiliations:** ^1^Institute of Immunity and Transplantation, UCL Division of Infection and Immunity, University College London, Royal Free Hospital, London, United Kingdom; ^2^School of Applied Science, Edinburgh Napier University, Edinburgh, United Kingdom

**Keywords:** regulatory T cells, gene therapy, immunotherapy, chimeric antigen receptor, T cell receptor, autoimmunity

## Abstract

Adoptive therapy with polyclonal regulatory T cells (Tregs) has shown efficacy in suppressing detrimental immune responses in experimental models of autoimmunity and transplantation. The lack of specificity is a potential limitation of Treg therapy, as studies in mice have demonstrated that specificity can enhance the therapeutic potency of Treg. We will discuss that vectors encoding T cell receptors or chimeric antigen receptors provide an efficient gene-transfer platform to reliably produce Tregs of defined antigen specificity, thus overcoming the considerable difficulties of isolating low-frequency, antigen-specific cells that may be present in the natural Treg repertoire. The recent observations that Tregs can polarize into distinct lineages similar to the Th1, Th2, and Th17 subsets described for conventional T helper cells raise the possibility that Th1-, Th2-, and Th17-driven pathology may require matching Treg subsets for optimal therapeutic efficacy. In the future, genetic engineering may serve not only to enforce FoxP3 expression and a stable Treg phenotype but it may also enable the expression of particular transcription factors that drive differentiation into defined Treg subsets. Together, established and recently developed gene transfer and editing tools provide exciting opportunities to produce tailor-made antigen-specific Treg products with defined functional activities.

## Introduction

Inherent checkpoints ensure that an immune response normally only occurs in response to genuine threats from pathogens. However, loss of this self-tolerance and resultant autoimmunity does occur, with prevalence as high as 12.5% in developed countries (1). The life-long chronic nature of both disease and treatment, and the high association of comorbidities (2) means the impact of autoimmunity on patients, their families, the health service, and the economy is substantial (3). The ultimate aim of autoimmune therapy would be to restore the lost self-tolerance while retaining the full potential of the immune system to respond to infection.

Regulatory T cells (Tregs) are an essential component of maintaining normal self-tolerance (4). Tregs possess powerful multifaceted suppressive mechanisms capable of controlling a broad range of innate and adaptive immune cells. Importantly, Treg-mediated suppression is exerted in a targeted antigen-specific manner, allowing for suppression of the immune response when appropriate without interfering with productive immunity when required (5). A number of approaches have been explored to boost Treg number and function in order to treat autoimmune disease. One of the most promising and actively explored of these at present is the adoptive transfer of Tregs. Augmenting Treg numbers by transferring an activated/expanded population of Tregs can ameliorate autoimmunity (6–8). However, the ability of disease-targeted Tregs to reverse ongoing autoimmunity, where high doses of polyclonal Tregs failed, is a strong indication that merely boosting numbers will not be sufficient to control disease (9–12). Appropriate disease-targeted antigen specificity is important to ensure that Tregs are localized and activated at the site of disease (13).

Achieving antigen specificity in a clinically applicable setting has been a major challenge in translating promising pre-clinical results to therapy. Treg specificity is determined by the T cell receptor (TCR) expressed on their surface. While it may be possible to expand the rare Treg clones with appropriate specificity to suppress disease, this process is prolonged, expensive, and has a number of conceptual issues, not least whether the appropriate disease-suppressing clones are present in autoimmune patients. To circumvent these problems, we, and others, have explored redirecting the specificity of bulk Treg populations by the gene transfer of a disease-relevant TCR (9). This process involves the genetic engineering of Treg with genes encoding TCR or chimeric antigen receptors (CARs) to target Treg specificity to antigens that are present at the sites of autoimmunity and absent in healthy tissues. This approach provides a mechanism to achieve disease-specific immune suppression while retaining systemic immune competence.

## Redirecting the Specificity of Tregs Using Gene Therapy

We were among the first laboratories to use TCR gene therapy to generate antigen-specific primary Treg with the capacity to mediate immune suppression *in vivo*. Murine CD4^+^ CD25^+^ Tregs were engineered to express a TCR that recognized a fragment of the ovalbumin (OVA) protein. When TCR-transduced Tregs were cultured with dendritic cells presenting OVA, engineered cells were capable of suppressing proliferation and IL-2 production by conventional T cells activated by a different antigen. These findings were validated *in vivo* using a model of autoimmune inflammatory arthritis showing that the presence of OVA, a non-disease causing antigen, in the knee was required for OVA-specific Treg to suppress inflammation caused by pathogenic T cells specific for arthritic antigens (9). The capacity of antigen-specific Treg to locally suppress pathogenic T cells with different specificities provides a strategy to treat autoimmune disease even when the target antigens that are recognized by the autoimmune T cells are unknown. Studies of human cells have shown that Tregs transduced with a TCR recognizing factor VIII, a clotting factor that often stimulates immune responses in hemophilia patients treated with recombinant protein, were able to suppress factor VIII-specific helper T cell responses (14). Similarly, TCR-transduced Treg specific for a pancreatic islet cell antigens were shown to suppress responses by pathogenic T cells with greater potency than polyclonal Treg *in vitro* (15).

As an alternative to the use of TCR gene transfer, a number of groups have explored transfer of CARs. CARs are a man-made alternative to TCR, made up of the antigen-binding domain of a specific antibody linked *via* an extracellular stalk to intracellular signaling motifs required for T cell activation. While TCR have the ability to recognize any cellular proteins when processed and presented by MHC molecules, CARs recognize only cell surface proteins. However, CARs have the advantage that recognition is independent of MHC and, therefore, applicable to patients irrespective of their MHC genotype. The intracytoplasmic portion of CARs contains signaling domains derived from molecules that are involved in T cell activation such as CD3ζ, CD28, 41BB, OX40, and others. In the setting of cancer immunotherapy, various combinations of signaling domains have been tested in second- and third-generation CAR constructs (16). At present, there is little experimental data about which combination of signaling domains may stimulate optimal Treg function, and it is not known whether anti-cancer effector T cells and suppressive Treg will require CARs with distinct intracellular signaling domains.

The efficacy of CAR-Treg has been demonstrated in studies of murine intestinal inflammation. Two groups have shown successful generation of CAR-Treg that maintain their phenotype when expanded, traffic to the gut and suppress inflammation in an antigen-dependent manner independent of MHC (17, 18). More recent studies have shown that factor VIII-specific human CAR-Treg function comparably to factor-VIII-specific TCR engineered Treg (19) and that human CAR-Treg specific for alloantigens can prevent graft rejection (20) and development of graft-versus-host disease (21) in xenogeneic transplantation models.

## Strategies to Identify the Most Appropriate Cell for Gene Engineering

It has become apparent that Treg heterogeneity extends beyond the well-defined thymic and peripherally induced subsets and represents populations of suppressive cells with multiple functions, niches, and genetic landscapes. FOXP3 is considered a master transcriptional regulator of Treg function because humans and mice that lack this gene also lack a functional Treg compartment and go on to develop an autoimmune-like disease (4). However, it has become clear that FOXP3 expression is not sufficient to imprint a stable and fully functional Treg phenotype. The discovery of 300 uniquely demethylated regions in Treg genes, known as the Treg-specific demethylated regions (TSDRs) offered fundamental insights into how a Treg phenotype is established. TSDRs were found to be specific to natural Treg (nTreg); the same markers were absent in *in vitro* generated induced-Treg, in FOXP3^+^ conventional T cells and in various helper T cell subsets (22, 23). This suggests that TSDRs have a Treg-specific role independent of FOXP3 expression. Interestingly, this TSDR profile was identified in a subset of cells from scurfy mice, a naturally occurring FOXP3-deficient strain, and it was found that these TSDR^+^ cells failed to suppress T cell responses *in vitro* but were less likely than TSDR^−^ cells to contribute to autoimmunity when adoptively transferred *in vivo* (24). Thus, a functional and stable Treg must express FOXP3 and have a distinct hypomethylation profile.

**Figure 1:**
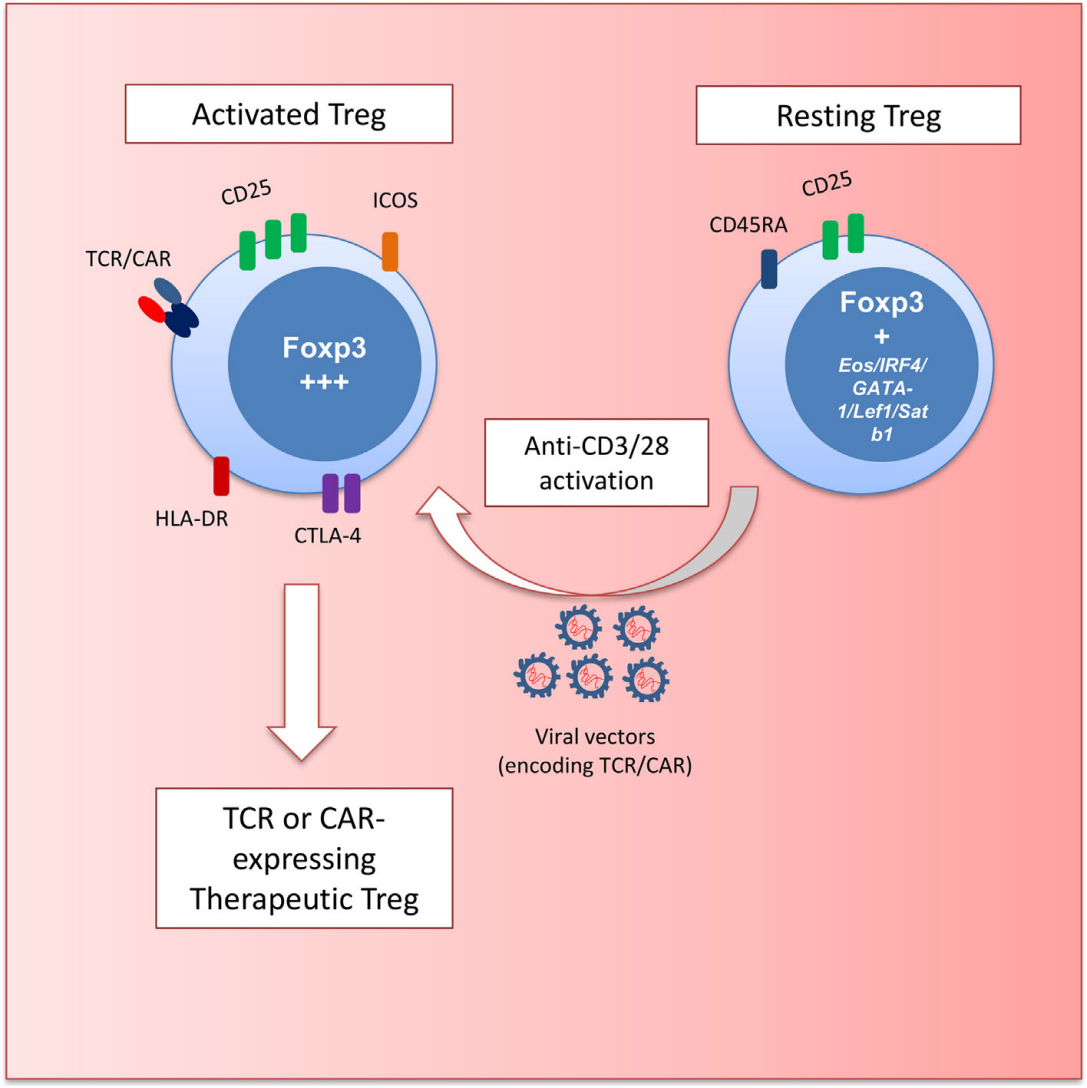
Schematic depicting regulatory T cell (Treg) gene engineering. Resting Treg (CD45RA^+^FOXP3^+^) are activated with anti-CD3 and anti-CD28 antibodies to transduce cells with retro- and lentiviral vectors encoding T cell receptors (TCRs) or chimeric antigen receptors (CARs). These engineered Treg have a defined specificity and an activated effector phenotype (HLA-DR^+^ICOS^+^CTLA-4^hi^CD25^hi^FOXP3^hi^) with potent suppressive potential.

Examination of Treg markers, function, and hypomethylation led to the identification of three subsets of FOXP3-expressing cells in the peripheral blood of humans (25) (Figure 1). FOXP3^hi^ CD45RA^−^ Tregs have been described as activated-Treg. These cells can be terminally differentiated and prone to apoptosis but are hypomethylated and highly suppressive. FOXP3^lo^ CD45RA^+^ cells are considered resting Treg. These cells also bear the Treg hypomethylation pattern and differentiate into an activated-Treg when stimulated. FOXP3^lo^ CD45RA^−^ cells are non-Tregs that do not display TSDR hypomethylation or suppressive function and produce inflammatory cytokines upon stimulation.

The most promising starting population for Treg engineering is the Foxp3-expressing CD45RA^+^ cells. These cells can efficiently expand *in vitro* (26) while maintaining their suppressive function (27). Currently used gene transfer protocols with retro- or lentiviral vectors involve stimulation with beads coated with anti-CD3 and anti-CD28 antibodies to trigger T cell proliferation that is required for efficient gene transfer (Figure 1). Thus, the ability of CD45RA^+^ Tregs to proliferate without losing functional activity provides a strong rationale for using these cells for genetic engineering.

In addition, use of rapamycin, an inhibitor of the mammalian target of rapamycin (mTOR) pathway, may improve the production of therapeutic Treg. Several publications have shown that rapamycin promotes the expansion of stable Treg subsets *in vitro*, maintaining hypomethylation at TSDRs over multiple rounds of expansion (28, 29). Data from our lab have previously shown successful reduction of mTOR activation in T cells engineered to express the proline-rich Akt substrate of 40 kDa (PRAS40), a negative regulator of the mTOR pathway (30). Genetic modification of Treg to express PRAS40 could be employed to ensure the maintenance of a Treg phenotype *in vivo*.

## Converting Conventional T Cells into Tregs through Gene Engineering

From the first reports describing Tregs that had been generated from conventional T cells *in vivo*, there have been attempts to replicate this for therapeutic use. The large pool of peripheral T cells makes the proposition of converting these cells into a population of suppressive cells attractive. Unlike protocols that use *in vitro* stimulation to induce Treg, gene therapy offers the prospect of converting cells into a stable population of “Treg-like” cells through genetic reprogramming.

We have previously shown that cotransfer of a FOXP3 gene construct with TCR can redirect the specificity and phenotype conventional T cells in mice (9). In these cells, expression of FOXP3 correlated with the upregulation of Treg-associated markers. Compared to conventional T cells transduced with TCR, cells transduced with TCR and FOXP3 were hypo-responsive to cognate peptide. Examination of suppressive function of these cells *in vitro* and *in vivo* showed that TCR plus FOXP3-converted T cells were able to suppress immune responses by T cells specific for a third party antigen, but that they were less potent than TCR-transduced nTreg.

The difference between engineered nTregs and FOXP3-converted T cells expressing the same TCR may lie in the requirement of non-FOXP3 factors to stabilize the Treg phenotype. Experiments in which conventional T cells were transduced with FOXP3 showed an induction of a partial Treg gene profile that could be stabilized by co-transfection with one of five transcription factors Eos, IRF4, GATA-1, Lef1, or Satb1 (31). It would be interesting to determine if the transfer of genes for FOXP3 plus one of the five transcription factors listed above would make the function of FOXP3-converted T cells comparable to TCR-transduced nTreg.

## Future Prospects: Engineering Disease-Specific Tregs

Heterogeneity of Treg is considered to be a relatively new finding but we have known for some time that there is a wide range of suppressive mechanisms utilized by Tregs that may be context dependent. Better understanding of the factors that mediate this heterogeneity could lead to the development of disease-specific Treg that target distinct inflammatory processes.

**Figure 2:**
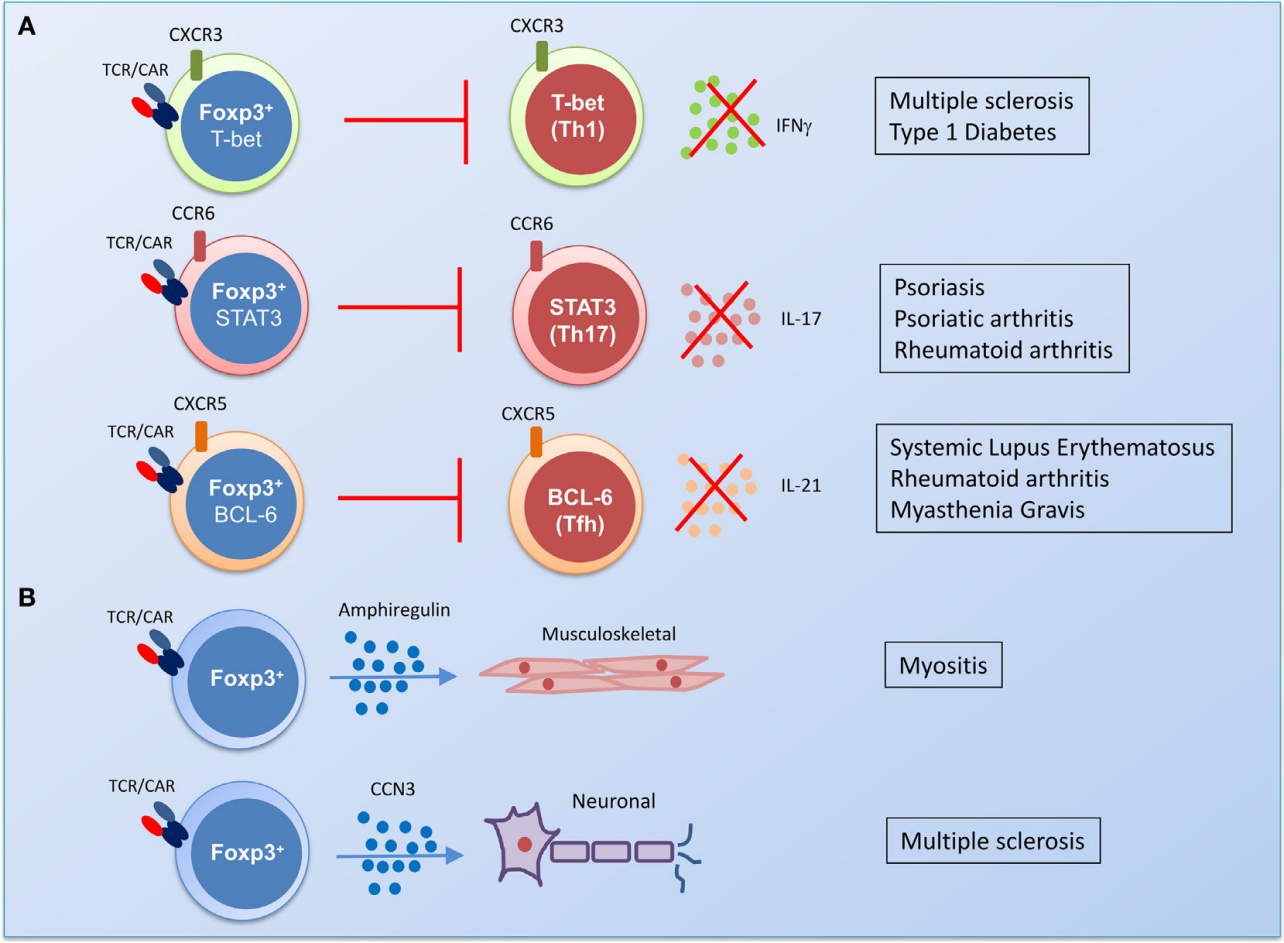
The potential of gene engineering to produce functionally specialized disease-suppressing regulatory T cells (Tregs). **(A)** The identification of transcription factors that drive differentiation of an effector Treg population in parallel with pathogenic T helper (TH) cells could be harnessed by gene therapy. In a predominantly TH1-driven chronic disease such as multiple sclerosis or type I diabetes, transduction of Treg with T cell receptor (TCR) or chimeric antigen receptor (CAR) and the transcription factor T-bet could generate antigen-specific Treg with the capacity to control TH1 responses *in vivo*. In rheumatic diseases, transduction of antigen-specific Treg with STAT3 could promote control of pro-arthritogenic TH17 responses. Antibody-driven diseases, such as systemic lupus erythematosus, rheumatoid arthritis, and myasthenia gravis, could be targeted by antigen-specific Treg that express the transcription factor associated with follicular helper T cells, BCL6. **(B)** Gene therapy could also be used to target the damage caused by chronic inflammation by transducing Treg with genes for factors that promote homeostatic tissue repair. Amphiregulin-producing Treg are enriched in the muscle and have been shown to promote repair of damaged tissue (38–40), while the production of the protein CCN3 by Treg has been shown to promote the repair of the myelin sheath in a mouse model of multiple sclerosis (41).

It is now clear that Treg undergo differentiation into an effector phenotype expressing distinct transcription factors, chemokine receptors, and displaying different antigen recall responses (32). T-bet-expressing Tregs differentiate in parallel with T helper (TH)1 cells; they express CXCR3 and are required for competitiveness at IFNγ-rich sites and for homeostasis of Treg in a model of TB infection. Similarly, in an experimental transplant model, IFNγ was required for promoting the survival of alloantigen-specific Th1-like Treg (33). Further studies have shown that upregulation of STAT3 expression is associated with increased CCR6 and the control of TH17 responses (34) and BCL6 expression is required for CXCR5 on Treg and suppression of follicular helper T cell responses (35–37). Thus, genetic engineering of Tregs with these transcription factors in the relevant autoimmune setting might facilitate successful trafficking of Treg to the site of inflammation and promote effective control of pathogenic responses (Figure 2).

However, a number of papers have highlighted that while Tregs expressing transcription factors associated with TH phenotypes are functional, these cells have a high proportion of inflammatory cytokine-producing T cells (32, 42, 43). Moreover, it has been suggested that these cells are not stable and that TCR stimulation can lead to their conversion into pathogenic effector cells (43, 44). Supporting this finding are data describing an increase in IFNγ-producing Tregs in patients with multiple sclerosis and type-1 diabetes. In these individuals, production of IFNγ by Tregs was associated with expression of T-bet and with an impaired suppressive function compared to non-IFNγ-producing cells (44, 45). Thus, the capacity of Treg to adopt an effector function could potentially contribute to pathogenesis.

Beyond the broad transcriptional approaches mentioned above, a more nuanced approach in which Treg are genetically modified to secrete regulatory effector molecules or made resistant to environmental signals that impair Treg function could contribute to the development of disease-specific therapy. In patients with rheumatoid arthritis, anti-TNF therapy has been incredibly effective in treating disease, response to this therapy is associated with the induction of Treg that can secrete IL-10 and TGFβ to control inflammatory responses (46). Using this insight into the mechanism of action of an established therapy, it might be possible to engineer Treg to secrete IL-10 and TGFβ, which could favor the suppression of arthritic inflammation. By contrast, a major concern of adoptive therapy with engineered Treg is the risk that an inflammatory environment *in vivo* may trigger the conversion into antigen-specific pathogenic effector T cells. To address this, newer technologies such as CRISPR could be used to undertake gene editing; making Treg impervious to inflammatory signals by editing out cytokine receptors such as IL-6 offers an attractive option for such an approach. Moreover, the propensity of Tregs from patients with autoimmunity to produce inflammatory cytokines could be ablated by using CRISPR to remove genes for IFNγ or IL-17 from engineered Treg. An equally novel approach might be to utilize features of tissue-resident Tregs that have recently been shown to contribute to tissue protection (47) and the repair of damaged tissue (38–41) *via* distinct transcription factors (Figure 2). This is a tantalizing glimpse of the additional potential of Treg therapy not just to suppress inflammation but to aid in repair of damaged tissue.

## Conclusion

The rapid development of technology that allows genetic engineering of primary immune cells has opened the door to a world of potential therapeutic interventions. The prospect of producing tailor-made cellular therapies with disease-specific function could revolutionize the treatment of autoimmunity.

## Author Contributions

JM and GW—research and writing of review, and critical revisions of the manuscript. HS—contributed to research and writing the review. All authors approved the manuscript before submission and agreed to be accountable for the content.

## Conflict of Interest Statement

All other authors declare no that the research was conducted in the absence of any commercial or financial relationships that could be construed as a potential conflict of interest. HS is scientific advisor and shareholder of Cell Medica and has shares in this biotech company.
